# Impact of spatial dispersion, evolution, and selection on Ebola Zaire Virus epidemic waves

**DOI:** 10.1038/srep10170

**Published:** 2015-05-14

**Authors:** Taj Azarian, Alessandra Lo Presti, Marta Giovanetti, Eleonora Cella, Brittany Rife, Alessia Lai, Gianguglielmo Zehender, Massimo Ciccozzi, Marco Salemi

**Affiliations:** 1 College of Public Health and Health Professions and College of Medicine, Department of Epidemiology, University of Florida, Gainesville, FL, USA; 2 Emerging Pathogens Institute, University of Florida, Gainesville, FL, USA; 3 Department of Infectious, Parasitic and Immunomediated Diseases, Istituto Superiore di Sanità, Rome, Italy; 4 Department of Pathology, Immunology and Laboratory Medicine, University of Florida, Gainesville, FL, USA; 5 Department of Biomedical and Clinical Science, Infectious Diseases and Immunopathology Section, ‘L. Sacco’ Hospital, University of Milan, Milan, Italy; 6 University Hospital Campus Bio-Medico, Italy

## Abstract

Ebola virus Zaire (EBOV) has reemerged in Africa, emphasizing the global importance of this pathogen. Amidst the response to the current epidemic, several gaps in our knowledge of EBOV evolution are evident. Specifically, uncertainty has been raised regarding the potential emergence of more virulent viral variants through amino acid substitutions. Glycoprotein (GP), an essential component of the EBOV genome, is highly variable and a potential site for the occurrence of advantageous mutations. For this study, we reconstructed the evolutionary history of EBOV by analyzing 65 GP sequences from humans and great apes over diverse locations across epidemic waves between 1976 and 2014. We show that, although patterns of spatial dispersion throughout Africa varied, the evolution of the virus has largely been characterized by neutral genetic drift. Therefore, the radical emergence of more transmissible variants is unlikely, a positive finding, which is increasingly important on the verge of vaccine deployment.

Ebola virus Zaire (EBOV), a member of the *Filoviridae* family, is a virulent Category A pathogen that causes considerable morbidity and mortality^1^. The EBOV genome is a linear, non-segmented, single-stranded RNA approximately 19,000 nucleotides long, coding for seven structural and one non-structural protein^2^. EBOV causes Ebola viral disease (EVD), characterized by fever, malaise, and other nonspecific symptoms such as myalgia, headache, vomiting, and diarrhea^3^. About 30%–50% patients manifest hemorrhagic symptoms^4^. Moreover, in some severe cases multi-organ dysfunction, including hepatic damage, renal failure, and central nervous system involvement occur, leading to shock and death^1^. The virus was first identified in 1976 during the epidemic of hemorrhagic fever in Zaire, now Democratic Republic of Congo (DRC), with the epicenter of the outbreak in Yambuku. EBOV appeared again in DRC in 1977 near Yambuku and subsequent outbreaks among humans have occurred in west-central Africa in distinct waves during 1994-1997 and 2001-2005^5^.

The recent and ongoing outbreak of EBOV began in February 2014 in forested areas of Southeastern Guinea, and cases have now been reported among several districts of Guinea. The 2014 EBOV outbreak has additionally been affecting the West African countries of Liberia, Nigeria, and Sierra Leone and have most recently spilled over to Europe and North America^6^. A total of 8,376 cases, with 4,024 deaths, have been reported to the World Health Organization (WHO) as of October 7, 2014^3^. On August 8, 2014, WHO declared the Ebola epidemic in West Africa a public health emergency of international concern.

The role of zoonosis in EBOV transmission is well recognized, yet incompletely understood. Between 2001 and 2005, 47 dead animals, including 23 great apes (18 gorillas, 5 chimpanzees), were discovered in Gabon and DRC. EBOV infection was confirmed for 13 gorillas, three chimpanzees, and one duiker^7^. The ongoing seventh DRC Ebola outbreak has been traced back to a single index case, a pregnant woman who butchered a monkey and subsequently spread the infection to four healthcare workers^8^. Fruit bats have also been implicated in harboring the virus^9^. However, the spatial dispersal of EBOV is incompletely explained by a single zoonotic transmission event. More likely, several animals are responsible for bridging transmission events between epidemic waves^10^.

With the current epidemic ongoing and new cases reported daily, there is an urgent need to understand the transmission of EBOV in Africa. This requires an understanding of geographic spread of the virus in the context of evolutionary factors (i.e. selection) driving the repeated emergence of EBOV in Africa, which can be inferred using historical genetic data from previous outbreaks. The spatial dispersal can also provide important insights that will allow for assessment of the impact of zoonosis on current and past epidemics. Previous phylogenetic analyses of the 2014 Ebola outbreak have focused on the phylogenetic relationships among strains isolated from Guinean patients and other sequences of the genus *Ebolavirus*, which includes *Bundingyo*, *Reston*, *Sudan*, *Tai Forest* and *Zaire* species^6^,^11^. Estimates of evolutionary rates have shown that the accumulation of nucleotide substitution has nearly doubled during the current epidemic when compared to previous outbreaks and resulted more frequently in non-synonymous polymorphisms^11^,^12^. These findings suggest that the ecological niche for Ebola may be expanding due to the emergence of new genetic variants driven by ongoing positive selection^13^. However, a systematic investigation of how selection has been affecting EBOV spatial dispersion across West Africa leading the past and current epidemic waves is still lacking. In this study, we used Bayesian phylogeography to reconstruct the spatial spread of the virus since the 1970s and analyzed the posterior distribution of phylogenetic trees for evidence of selection along the major backbone of the EBOV genealogies – representing major lineages propagating through time from root node to each epidemic outbreak. The results clearly indicate that EBOV evolution has consistently been driven by neutral genetic drift, rendering the emergence of even more aggressive variants under positive selection unlikely.

## Results

**Figure 1:**
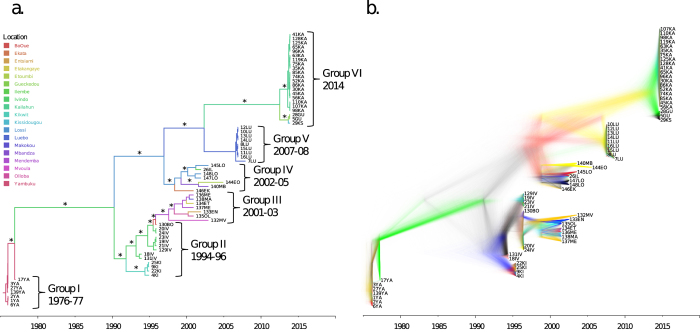
Bayesian MCC genealogy and DensiTree representation of posterior distribution of trees from Bayesian phylogenetic analysis. (**a**) Bayesian maximum clade credibility (MCC) tree of EBOV GP gene with branch lengths scaled in time by enforcing a relaxed molecular clock. Strain labels are colored to indicate the sampling location according to the legend in the figure. Branches labeled with an asterisks are well supported, having a posterior probability >0.85. (**b**) A posterior distribution of trees was obtained from Bayesian phylogenetic analysis of EBOV GP gene using GMRF Skygrid model and relaxed molecular clock as implemented in BEAST v1.8.0. Tip dates for each node represent the year of isolate collection. DensiTree provides a visualization of the posterior distribution of trees and the frequency of node clustering for support of clades in the overall topology. Branches are colored based on distinct clades. Well-supported branches are indicated by solid colors whereas webs represent little agreement.

EBOV epidemics were divided into groups based on the temporality of each epidemic wave and phylogenetic clustering in the Bayesian analysis (Fig. 1A). The genealogy and spatial dispersion was mapped for visualization. The Bayesian Maximum Clade Credibility (MCC) EBOV phylogenetic tree exhibited staircase-like topology with focal epidemics in specific geographic areas giving rise to epidemics in subsequent years and leading to the current 2014 epidemics (Fig. 1A). Furthermore, the genealogy illustrated the emergence of two lineages after the 1976 epidemic in DRC. The first lineage gave rise to the 1994-1996 epidemics in Gabon and DRC (Group II), and later to 2001-2002 epidemics in Gabon, Cameroon, and Republic of Congo (Group III). The second lineage gave rise to the 2001-2005 epidemics in Gabon and Republic of Congo (Group IV). Group IV sequences included EBOV sequences collected from humans and great apes that were concurrently infected, highlighting the spillover events that occurred during this time point. Last, Group VI represents the current 2014 epidemic in Guinea (Gueckedou and Kissidougou) and Sierra Leone (Kailahun District), which emerged from a shared common ancestor with sequences from the 2007 epidemic in Luebo, DRC (Group V). The general topologies in the posterior distribution of trees (Fig. 1B) were highly consistent with the topology of the MCC tree (Fig. 1A), indicating that this particular topology was well supported given the data.

**Figure 2:**
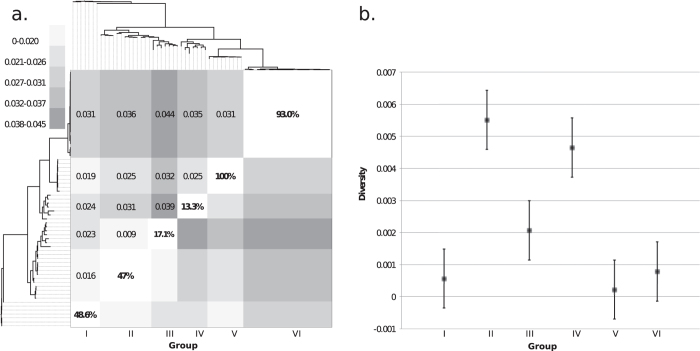
Comparison of between and within group evolutionary diversity with phylogenetic signal. (**a**) Between group estimates of evolutionary diversity (i.e. nucleotide substitutions per site from averaging over all sequence pairs between groups) using maximum composite likelihood model with a gamma distribution. Each group represents a distinct group (I - VI) defining a specific epidemic. Percentages along the diagonal represent the results from likelihood mapping phylogenetic signal analysis (material and methods). The greater the percentage, the higher the “star-like” signal for each clade. (**b**) Within group estimates for evolutionary diversity and standard errors using the maximum composite likelihood model with a gamma distribution for six distinct groups representing distinct epidemics.

**Figure 3:**
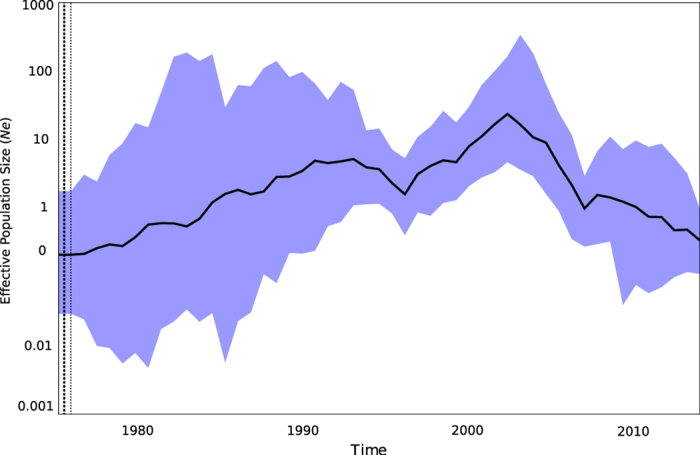
Effective population size (*Ne*) estimates from Bayesian phylogenetic analysis. The solid black line and the shaded blue upper and lower bounds represent, respectively, median and 95% high posterior density (95% HPD) intervals estimates of *Ne* over time. *Ne* values were estimated in BEAST package v1.8.0 employing a non-parametric Gaussian Markov randomfield (GMRF) Skygrid evolutionary model assuming a relaxed clock.

The phylogenetic structure between distinct epidemics was further investigated by likelihood mapping analysis^14^. The evaluation of all possible quartets (group of four sequences) for each group of sequences sampled during distinct EBOV epidemic waves (Fig. 1) demonstrated significant differences in phylogenetic signal between epidemics (Supplementary Fig. S1). Most notably, 100% and 93% of the quartets from the 2007 epidemic in Luebo, DRC (Fig. 1 - Group V) and the current 2014 epidemic in Sierra Leone and Guinea (Fig. 1 - Group VI), respectively, were distributed in the center of the likelihood map (Fig. 2A). This indicates strong star-like phylogenetic signal reflecting an outburst of new lineages due to exponential epidemic spread.^15^ Interestingly, however, the current 2014 outbreak (group VI), as well as the 1976-77 (group I) and 2007-08 epidemics (group V) were characterized by the lowest genetic diversity (Fig. 2B). In contrast, groups II – IV, including strains from the 1990s and early 2000s epidemics, were characterized by more structured tree-like topology (star-like signal <50%), and higher within groups diversity in case of group II-IV (Fig. 2B), typical of multiple introductions of genetically diverse strains evolving from independent lineages over a longer time span. The overall demographic history of EBOV was also investigated through a Bayesian coalescent framework^16^. The GMRF Skygrid model – representing non-parametric estimates of virus effective population size (*Ne*) over time – with a relaxed molecular clock was found to be the best fitting model. Molecular clock calibration estimated the evolutionary rate of the EBOV GP gene at 1.075 × 10^−3^ substitutions site^−1^ per year (95% HPD 1.22 × 10^−3^, 9.32 × 10^−4^) and dated the time of the most recent common ancestor (TMRCA) as 1976 (95% HPD 1975-1976), in agreement with known epidemiological data^5^. *Ne* estimation (i.e. number of genomes effectively giving rise to the next generation) indicated an increasing population that peaked in 2007 and subsequently decreased thereafter (Fig. 3), consistent with the decrease in genetic diversity observed during the current epidemic.

Phylogeographic analysis illustrated the spatial diffusion of EBOV within the West-African region (Supplementary File 1). All geographical migrations were found to be significant (lnBF > 3) when the dispersal was assessed using SPREAD. From our analysis, it is likely that migration of EBOV outward from Yambuku, DRC, in 1976 served to seed additional regions in DRC and Gabon where the virus emerged again in 1994 and 1995, respectively. This lineage then radiated outward causing sporadic outbreaks in Gabon, Cameroon, and Republic of Congo from 2001 to 2002. A similar radiating spatial dispersion pattern was inferred among Gabon and Republic of Congo strains (Group IV) causing contemporaneous human and non-human primate infections. A closely related virus emerged again in Luebo, DRC, in 2007, and subsequently led to the current Sierra Leone and Guinea epidemics 2,400 miles away. Interestingly, the Republic of Congo and Gabon epidemics spanning 2001 to 2005 (Groups III and IV) are geographically compartmentalized, representing two distinct clades on separate lineages of the MCC genealogy.

**Figure 4:**
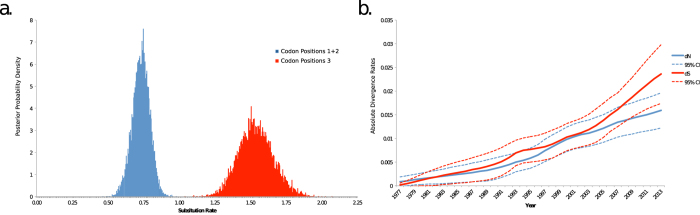
EBOV absolute non-synonymous and synonymous divergence over time. (**a**) Posterior probability density of evolutionary rates for 1 + 2 (blue) and 3^rd^ (red) codon positions (**b**) Mean non-synonymous (dN) and synonymous (dS) divergence along backbone branches from a sub-sample of trees from the posterior distribution during the Ebola epidemics are represented by blue and red lines, respectively. Dashed lines represent one standard deviation above and below mean dN or dS. The y-axis represents the number of nucleotide changes per site.

Given the observed variations in spatial dispersion patterns and evolutionary dynamics between different epidemics, we then sought to investigate the presence of selective pressures driving the emergence of new viral variants during each epidemic wave. We first assessed synonymous (dS) and non-synonymous (dN) substitutions among sequence pairs within groups I-VI. While dN/dS increased among groups along the backbone of the genealogy leading to the 2014 epidemic, only the 1976-1977 (Group I) significantly departed from strict neutrality (i.e., dN > dS) (Supplemental Table 1). A greater number of dN mutations was also observed for the 2014 epidemic (Group VI). As these findings provided evidence that dN and dS varied across EBOV epidemics, we then compared rates of change at the codon level that would result in dN or dS substitutions by independently estimating the evolutionary rate of the 1^st^ + 2^nd^ codon position and 3^rd^ codon position. Overall, the evolutionary rate (molecular clock) for the 3^rd^ codon position [1.54 (95% HPD 1.32, 1.79)] was significantly greater than the 1^st^ + 2^nd^ codon position [0.73 (95% HPD 0.60, 0.84)], which suggests low levels, if any, of positive selection (Fig. 4A). Comparison of absolute rates of dS and dN substitutions along the backbone path of the EBOV genealogy also showed that dS and dN substitutions accumulated at similar rates (Fig. 4B), indicating that the evolution of the major of EBOV lineage successfully propagating through time, from its emergence in 1976 until the current epidemic, has been driven by neutral genetic drift. Selection was further investigated by evaluation of specific amino acid sites along internal and terminal braches of the genealogy using six methods available on the online server ( http://www.datamonkey.org)^17^. The analysis identified only one amino acid site (429) that was found to experience significant diversifying selection by three methods, and two sites (210 and 664) under significant purifying selection by three and four methods respectively (Table 1). When assessing the location of these sites along the branches of the genealogy, however, we found that although amino acid site (429) was located on an internal branch, it did not propagate along the backbone of the lineage leading to the current epidemic. Remaining mutations were located on terminal branches of the genealogy, likely representing transient polymorphisms.

## Discussion

At the time of this writing, the countries of Guinea, Liberia, and Sierra Leone have all reported intense, widespread transmission of EBOV, whereas others have had an initial case or cases with localized transmission (Nigeria, Senegal, Spain, and the United States of America). The phylogenetic history of EBOV leading to the current epidemics in Africa are seemingly complex, with punctuated focal outbreaks emerging in multiple discrete geographic regions over the past four decades. Upon analysis of 65 dated EBOV GP gene sequences from several affected countries throughout West Africa, we found that the current Sierra Leone epidemic in Kailahun is most closely related to the circulating viral strain in the Guinean cities of Kissidougou and Gueckedou (Fig. 1), representing two distinct clades in the Bayesian MCC genealogy. The single clade including all Kailihun cases is consistent with a single point source introduction followed by direct person-to-person transmission. This is concordant with previous analyses of the current Guinea outbreak, which found that the Guinean epidemic strain emerged from an EBOV lineage previously found in DRC, Republic of Congo, and Gabon^6^,^11^. Furthermore, the two lineages responsible for the current Guinea and Sierra Leone outbreaks diverged from EBOV responsible for the 2007 outbreak in Luebo, DRC. This divergence between the Guinean epidemic from the Central African lineage has previously been estimated to have occured in 2002^11^.

The spread of EBOV from Central to West Africa may involve movement of people and animals, increasingly connected by expanding populations and improved infrastructure (i.e. roads). Pigott *et al.* (2014) recently estimated that zoonotic transmission is possible in 22 countries of Central and West Africa, encompassing a population greater than 22 million^13^. In comparison to previous Central Africa outbreaks in Congo, 1995 and Uganda, 2000, recent studies have demonstrated that the transmission dynamics of the current epidemics are comparable in terms of reproductive rate, serial interval, and case fatality ratio^18^. However, EBOV transmission dynamics in Central and West African are seemingly different. In West Africa, villages are linked by an extensive transportation network, whereas in Central Africa the affected villages are remote and poorly connected^8^. Therefore, the dissemination patterns may vary based on geography, in concert with the epizootic epidemiology of EBOV in and among animal vectors. Furthermore, previous EBOV spread rates have been estimated at 50 km per year^10^, which complicates the explanation of the nearly 3,900 km traversed in the migration from Central to West Africa over a seven year period. While the introduction into West Africa has yet to be explained, it is evident that there are strong epizootic and zoonotic components to EBOV transmission. For example, the Republic of Congo and Gabon epidemics spanning 2001 to 2005 (Groups III and IV) are phylogeographically distinct. Notably, Group IV (Fig. 1A) contains both human and great ape strains from Republic of Congo and Gabon epidemics on a single, well-supported monophyletic clade with high within-group diversity, suggesting contemporaneous epidemics in humans and animals and multiple non-human-primate-to-human spillover events^7^,^19^. The compartmentalization of these epidemics suggests variations in reservoirs and intermediate non-human primate host species, as has been previously suggested^5^.

GP is the most widely studied region of the EBOV genome, having an evolutionary rate ideal for phylogenetic studies of this timescale^20^,^21^. Its expression on the virion surface is responsible for host cell attachment and fusion, making it an essential component of pathogenicity as well as a potential vaccine target^22^,^23^. Yet, few studies have comprehensively assessed selection in the GP gene^24^. Our estimate of the evolutionary rate of the GP gene was 1.075 × 10^−3^ substitutions site^−1^ per year (95% HPD 1.22 × 10^−3^, 9.32 × 10^−4^), consistent with previous studies^11^, and *Ne* reconstruction demonstrated an exponentially increasing population. Despite varying patterns of spatial dispersal and epidemic origin, the central finding of the current study is that EBOV evolution, at least after the first known epidemic wave in 1976-77, has largely been driven by neutral genetic drift. There was some evidence that dN/dS has varied across epidemic waves but, excluding the initial 1976-77 epidemic when there were no observed dS substitutions, deviations from neutrality were not statistically significant. Furthermore, amino acid substitutions were mostly transient, either located on terminal branches of the genealogy or removed by purifying selection. Investigation of site-specific selection only identified one site on an internal branch that was significant for positive selection. Importantly, however, no amino acid substitutions were located on backbone branches leading to the current 2014 epidemic. Overall, the rates of dN and dS did not significantly vary between epidemic waves, providing little evidence that selection acting upon the GP gene is driving EBOV evolution. Given that we observed a greater proportion of dN than dS substitutions within the 2014 clade (Group VI), we cannot exclude the possibility that advantageous mutations could occur in the future and become fixed in the population. Yet, it is noteworthy that while clades with dN/dS > 1 were observed during past epidemics, these variants did not propagate through time.

The proline to leucine substituion at amino acid site 429 in the Mucin-like domain (MLD, nucleotides 1,285-1,287) occurred twice along the EBOV genealogy, once after the bottleneck following Group I, with the diverging Groups II and III possessing the substitution, and again along the branch leading to the current 2014 epidemic. Interestingly, there was a reversion of leucine to proline on a terminal branch of Group II (strain 4KI). Although the MLD is dispensable for EBOV infections *in vitro*^25^,^26^ and is the least conserved of the GP domains^27^ it has been determined to have several functions, including enhancing viral adhesion^28^,^29^ and protecting conserved regions of GP from antibody recognition^30^,^31^. Therefore, the potential effect of this mutation on virulence and/or pathogenicity should be further investigated. Unfortunately, we were unable to identify the specific location of this amino acid and the potential effect of its change on local structure because the crystal structure of this highly flexible domain has not yet been determined. Meanwhile, the two sites found to be under purifying selection were located on terminal branches of the genealogy. Overall, together with the observation of equivalent increases in dN and dS across the genealogy, it is evident that EBOV evolution is largely driven by neutral genetic drift. Furthermore, while it has been observed that the rate of dN is higher among EBOV genomes in the current epidemic, there is no evidence yet that any of these mutations are adaptive and/or if they will become fixed in the population or removed by purifying selection. Based on the current analysis, we posit the latter scenario is more likely^12^.

The ongoing EBOV epidemics in Central and West Africa continue to claim lives, and it is estimated that the epidemics will expand exponentially^12^. As the transmission dynamics of these epidemics are consistent with those previously studied, our analysis provides evidence that the exponential growth of cases is attributed to variations in population structure (e.g., mobility) and large-scale transmission events, rather than viral factors such as enhanced virulence or transmissibility. Phylogenetic analysis of the epidemic has revealed several gaps in our knowledge in Ebola epidemiology. First, it is evident that the virus has been circulating in animal populations spanning the 2007 and 2014 epidemics. More studies are needed to examine zoonotic transmission and subsequent spillover into human populations, which would better explain the spatial rate of spread and the bottlenecks observed between epidemics. Second, while EBOV evolution has until now been characterized by neutral genetic drift, the progressive accumulation of mutations during epidemic spread increases the possibility of viral adaptation. Overall, continued genomic surveillance of the epidemic is required to assess adaptability and selection, which is increasingly important on the verge of vaccine deployment.

## Material and methods

### Sequence data set

All available Zaire Ebola Virus (EBOV) GP gene sequences, with known sampling date and geographical location, were obtained from the National Center for Biotechnology Information ( http://www.ncbi.nlm.nih.gov/). Due to the overrepresentation of EBOV dated sequences from Sierra Leone, a randomization was performed to reduce the number of taxa and unbias the phylogeographical reconstruction. Moreover, three partial GP sequences were excluded from the dataset. The final dataset included 65 dated GP gene sequences with known geographical location. The sampling dates ranged from 1976 to 2014. The sampling locations were Guinea (Gueckedou, n = 2 and Kissidougou, n = 1), Gabon (Ivindo, n = 9; BoOue, n = 1; Mendemba, n = 2; Makokou, n = 1; Etoumbi, n = 1; Ekata, n = 1), DRC (Yambuku, n = 7; Kikwit, n = 4; Luebo, n = 9), Congo (Mvoula, n = 1; Entsiami, n = 1; Etakangaye, n = 1; Olloba, n = 1; Mbandza, n = 1; Lossi, n = 3); and Sierra Leone (Kailahun, n = 19). This dataset was used to estimate the evolutionary rate, perform the time-scaled phylogenetic and phylogeographic analysis, and assess selection. All sequences were aligned using ClustalX software and edited by using Bio-Edit software v.7.0, as previously described^32^. jModelTest 2 was used to select the simplest evolutionary model that adequately fit the sequence data^33^.

### Likelihood mapping analysis and diversity estimates

In addition to testing for phylogenetic signal, the analysis of groups of four randomly chosen sequences (quartets) through the process of likelihood mapping provides an indication of phylogenetic structure and population expansion^14^. For a quartet, three unrooted tree topologies are possible. The likelihood of each topology is estimated and the three likelihoods are reported as a dot in an equilateral triangle (the likelihood map). Three main areas in the map can be distinguished: the three corners representing fully resolved tree topologies (i.e., the presence of tree-like phylogenetic signal); the center, which represents star-like genealogy, and the three areas on the sides indicating network-like genealogy (i.e., presence of recombination or conflicting phylogenetic signals). Extensive simulation studies have shown that >50% dots falling within the central area indicate substantial star-like signal, or outburst of multiple phylogenetic lineages, often associated with exponential epidemic growth^14^,^15^. Likelihood mapping analysis was performed using the program TREE-PUZZLE^34^ by analyzing all possible quartets for each of the six groups representing specific epidemics. The analysis was then repeated for the entire genealogy. Between and within group divergence/diversity were determined by estimating the nucleotide substitutions per site over all sequence pairs between and within groups using MEGA v6.06^35^. A maximum composite likelihood model with gamma distribution was used to estimate divergence and diversity for each of the six distinct clades/groups.

### Evolutionary rate estimates

Evolutionary rates were estimated using a Bayesian Markov chain Monte Carlo (MCMC) method implemented in BEAST package v1.8.0^16^,^36^ employing a non-parametric Gaussian Markov randomfield (GMRF) Skygrid^16^,^37^,^38^ evolutionary model and both a strict and relaxed clock with an uncorrelated log normal rate distribution. The GMRF Skygrid model provides enhanced performance compared to Bayesian skyline plot (BSP) and Bayesian Skyride models by parameterizing *Ne* and smoothing the trajectory. TMRCA is also better estimated since the prior is independent of the genealogy. The alignment was partitioned into first + second codon positions and third codon positions. An HKY nucleotide substitution model with gamma-distributed rates among sites was selected. Chains were conducted for 2 × 10^8^ generations sampled every 20,000 steps for each molecular clock model. Posterior probabilities were calculated using the program Tracer v. 1.6 after 10% burn-in. Convergence was assessed on the basis of the effective sampling size (ESS) and only parameter estimates with ESS values of >200 were accepted. Marginal likelihoods estimates for each model were obtained using path sampling and stepping stone analyses^39^,^40^,^41^. Uncertainty in the estimates was indicated by 95% highest posterior density (95% HPD) intervals, and the best fitting models were selected by a Bayes Factor^39^,^42^. The GMRF Skygrid model enforcing a relaxed molecular clock was selected as the most appropriate representation of the Ebola demographic history^42^,^43^,^44^.

### Time-scaled phylogeography reconstruction

The time-scaled phylogenetic reconstruction and the phylogeographic analysis were conducted by using a Bayesian MCMC method implemented in BEAST package v1.8.0^36^,^41^ implementing the HKY+G nucleotide substitution model with codon partitioning and assuming a relaxed clock with an uncorrelated log normal rate distribution and the GMRF Skygrid demographic model (previously selected by a Bayes factor). Statistical support for specific clades was obtained by calculating the posterior probability of each monophyletic clade. MCMC chains were run for at least 200 million generations and sampled every 20,000 steps. The continuous time MCMC process over discrete sampling locations implemented in BEAST v1.8.0^45^ was used for the phylogeographic analysis by implementing the Bayesian Stochastic Search Variable Selection (BSSVS) model, which allows diffusion rates to be zero with a positive prior probability. Comparison of the posterior and prior probabilities of the individual rates being zero provided a formal BF for testing the significance of the linkages between locations. Rates with a lnBF of >3 were considered well supported and formed the migration pathway. The maximum clade credibility (MCC) tree (the tree with the largest product of posterior clade probabilities) was selected from the posterior tree distribution after a 10% burn-in using TreeAnnotator v1.8.0. The final trees were manipulated in FigTree v1.4.2 for display purposes. The posterior distribution of trees was also visualized with DensiTree^46^. In the DensiTree, well-supported branches are designated by solid colored areas, while webs represent less agreement. The migration routes were visualized using SPREAD v1.0.6 and mapped with ArcGIS v10.1.

### Selection analysis

MEGA6 was used to estimate the mean synonymous (dS) and nonsynonymous (dN) substitutions for each group of sequences representing distinct epidemic waves based on phylogenetic and epidemiological data^47^. For each group of sequences, the Nei-Gojobori method was used to test the hypothesis of dN > dS (*i.e.* a deviation from strict neutrality) and variance was computed using 1000 bootstrap replicates^48^. The number of dS and dN substitutions along the EBOV genealogy were then estimated by reconstructing and comparing ancestral sequences along the EBOV trees sampled from the posterior distribution from the Bayesian analysis. This approach is an empirical extension of the coalescent-based Bayesian molecular clock models^49^. Branch lengths proportional to either dS or dN substitutions were re-estimated using a subsample of 200 trees randomly selected from the posterior distribution obtained by BEAST and dN and dS point estimates over time were plotted. For each clock-like tree, estimates were obtained for all, internal, and backbone paths. The backbone path is the lineages effectively surviving from the root node to sequences sampled at the last time point. Sites under diversifying or purifying selection were assessed using six varying methods available on the Datamonkey online server ( http://www.datamonkey.org)^17^. Selection tests included Single likelihood ancestor counting (SLAC), fixed effect likelihood (FEL), internal FEL (IFEL), random effects likelihood (REL), Fast Unconstrained Bayesian Approximation for Inferring Selection (FUBAR), and Mixed Effects Model of Evolution (MEME)^50^,^51^,^52^. FEL and IFEL assess selection along all branch and internal braches respectively, while REL replicates the Nielson and Yang model implemented in PAML^53^. Site found to be statistically significant for positive or negative selection (P < 0.1, posterior probability >0.9, or Bayes Factor >50) by more than two methods were further assessed to identify their location within the EBOV genealogy.

## Author Contributions

T.A. conducted analysis, interpreted results, and prepared the manuscript; A.L.P. conducted analysis and prepared the manuscript; M.G.E.C., A.L. and G.Z. prepared the data sets, conducted analysis and prepared the manuscript; BR interpreted the results and prepared the manuscript; M.C. and M.S. served as senior authors, conceived the study, interpreted results, and prepared the manuscript.

## Additional Information

**How to cite this article**: Azarian, T. *et al.* Impact of spatial dispersion, evolution, and selection on Ebola Zaire Virus epidemic waves. *Sci. Rep.* **5**, 10170; doi: 10.1038/srep10170 (2015).

## Supplementary Material

Supplementary Video

Supplementary Information

## Figures and Tables

**Table 1:** Sites under diversifying and purifying selection based on analysis of Ebola glycoprotein gene.

	**SLAC**	**FEL**	**IFEL**	**REL**	**FUBAR**	**MEME**
Positive	—	**429**	—	170, 254, 330, 335, 367, 370, 376, 377, 388, 412, **429**, 442, 481, 524, 542	**429**	387, 388
Negative	**664**	5, 41, 173, **210**, 265, 268, 437, 495, 496, 511, 557, **664**	**210**, 495, 511, **644**	—	210, 374, **664**	—

Selection in the Ebola virus genealogy was assessed using the Datamonkey webserver by the following methods: SLAC, Single likelihood ancestor counting; FEL, fixed effect likelihood; IFEL, internal FEL; REL, random effects likelihood; FUBAR, Fast Unconstrained Bayesian Approximation for Inferring Selection, MEME, Mixed Effects Model of Evolution. Sites in bold were found to be under selection by two or more methods.
