# Supplementary material for: Impact of spatial dispersion, evolution, and selection on Ebola Zaire Virus epidemic waves
Source: Sci Rep. 2015 May 14;5:10170. doi: 10.1038/srep10170 (PMC4431419; doi:10.1038/srep10170)
Supplement: Supplementary Information [file srep10170-s2.pdf]

Impact of spatial dispersion, evolution, and selection on Ebola Zaire Virus epidemic waves

Taj Azarian[1,2,Ŧ], Alessandra Lo Presti[3,Ŧ], Marta Giovanetti[3], Eleonora Cella[3], Brittany Rife[4], Alessia Lai[5], Gianguglielmo Zehender[5], Massimo Ciccozzi [3,6*]; Marco Salemi[4,2*]

*Drs. Cicozzi and Salemi serve as co-senior authors; Dr. Salemi serves as corresponding author.
Ŧ Taj Azarian and Alessandra Lo Presti would like to be recognized as co-first authors

1. College of Public Health and Health Professions and College of Medicine, Department of Epidemiology, University of Florida, Gainesville, FL, USA; 2. Emerging Pathogens Institute, University of Florida, Gainesville, FL, USA; 3. Department of Infectious, Parasitic and Immunomediated Diseases, Istituto Superiore di Sanità, Rome, Italy; 4. Department of Pathology, Immunology and Laboratory Medicine, University of Florida, Gainesville, FL, USA; 5. Department of Biomedical and Clinical Science, Infectious Diseases and Immunopathology Section, 'L. Sacco' Hospital, University of Milan, Milan, Italy; 6. University Hospital Campus Bio-Medico, Italy

Author Contact

Massimo Ciccozzi
Viale Regina Elena, 299, 00161 Rome, Italy
Tel +39-06-49903187
massimo.ciccozzi@iss.it

#Marco Salemi
PO Box 103633, Gainesville, FL 32610, USA.
Tel/Fax +1 352 273 9567/8284
salemi@pathology.ufl.edu

*Corresponding Authors: Marco Salemi, Ph.D. - salemi@pathology.ufl.edu, Massimo Ciccozzi - massimo.ciccozzi@iss.it

Condensed Title: Phylogeography of Ebola Zaire Virus

Supplementary Table 1 - List of Ebola virus glycoprotrein (GP) sequences by year and location of collection

Name	Year	City/Village	Country	Latitude	Longitude	Accession	Source
1YA	1976	Yambuku	Democratic Republic of Congo	2°49'34.6"N	22°13'34.2"E	AF086833.2	Human
2YA	1976	Yambuku	Democratic Republic of Congo	2°49'34.6"N	22°13'34.2"E	AF272001.1	Human
3YA	1976	Yambuku	Democratic Republic of Congo	2°49'34.6"N	22°13'34.2"E	AY142960.1	Human
6YA	1976	Yambuku	Democratic Republic of Congo	2°49'34.6"N	22°13'34.2"E	EU224440.2	Human
27YA	1976	Yambuku	Democratic Republic of Congo	2°49'34.6"N	22°13'34.2"E	KC242801.1	Human
139YA	1976	Yambuku	Democratic Republic of Congo	2°49'34.6"N	22°13'34.2"E	U81161.1	Human
17YA	1977	Yambuku	Democratic Republic of Congo	2°49'34.6"N	22°13'34.2"E	KC242791.1	Human
18IV	1994	Ivindo	Gabon	0°09'24.2"S	12°10'22.6"E	KC242792.1	Human
131IV	1994	Ivindo	Gabon	0°09'24.2"S	12°10'22.6"E	U77384.1	Human
4KI	1995	Kikwit	Democratic Republic of Congo	5°02'00.0"S	18°49'00.0"E	AY354458.1	Human
9KI	1995	Kikwit	Democratic Republic of Congo	5°02'00.0"S	18°49'00.0"E	JQ352763.1	Human
22KI	1995	Kikwit	Democratic Republic of Congo	5°02'00.0"S	18°49'00.0"E	KC242796.1	Human
25KI	1995	Kikwit	Democratic Republic of Congo	5°02'00.0"S	18°49'00.0"E	KC242799.1	Human
19IV	1996	Ivindo	Gabon	0°09'24.2"S	12°10'22.6"E	KC242793.1	Human
20IV	1996	Ivindo	Gabon	0°09'24.2"S	12°10'22.6"E	KC242794.1	Human
21IV	1996	Ivindo	Gabon	0°09'24.2"S	12°10'22.6"E	KC242795.1	Human
23IV	1996	Ivindo	Gabon	0°09'24.2"S	12°10'22.6"E	KC242797.1	Human
24IV	1996	Ivindo	Gabon	0°09'24.2"S	12°10'22.6"E	KC242798.1	Human
129IV	1996	Ivindo	Gabon	0°09'24.2"S	12°10'22.6"E	HQ849547.1	Human
130BO	1996	BoOue	Gabon	0°05'37.6"S	11°56'57.0"E	AY058898.1	Human
134ET	2001	Etakangaye	Gabon	1°01'00.0"N	13°58'00.0"E	AY526100.1	Human
135OL	2001	Ollaba	Gabon	0°40'42.2"N	14°10'39.3"E	AY526099.1	Human
136ME	2001	Mendemba	Gabon	0°45'23.9"N	14°04'43.0"E	AY526098.1	Human
137ME	2001	Mendemba	Gabon	0°45'23.9"N	14°04'43.0"E	AY526105.1	Human
138MA	2001	Makokou	Gabon	0°34'00.0"N	12°52'00.0"E	AY526101.1	Human
146EK	2001	Ekata	Gabon	0°40'37.4"N	14°17'20.5"E	EU051632.1	Gorilla
26IL	2002	Ilembe	Gabon	29°22'59.3"S	31°10'16.7"E	KC242800.1	Human
133EN	2002	Entsiami	Republic of Congo	00°72'29"N	14°13'40"E	AY526102.1	Human
147LO	2002	Lossi	Republic of Congo	0°10'57.5"N	14°31'09.1"E	EU051631.1	Gorilla
148LO	2002	Lossi	Republic of Congo	0°10'57.5"N	14°31'09.1"E	EU051630.1	Gorilla
132MV	2003	Mvoula	Cameroon	2°51'07.7"N	11°35'09.3"E	AY526104.1	Human
140MB	2003	Mbandza	Republic of Congo	1°29'06.32"S	16°10'37.95"E	EU051635.1	Human
145LO	2003	Lossi	Republic of Congo	0°10'57.5"N	14°31'09.1"E	EU051633.1	Chimpanzee
144ET	2005	Etoumbi	Republic of Congo	0°01'13.0"N	14°53'21.3"E	EU051634.1	Human
8LU	2007	Luebo	Democratic Republic of Congo	5°21'00.0"S	21°25'00.0"E	HQ613403.1	Human
10LU	2007	Luebo	Democratic Republic of Congo	5°21'00.0"S	21°25'00.0"E	KC242784.1	Human

11LU	2007	Luebo	Democratic Republic of Congo	5°21'00.0"S	21°25'00.0"E	KC242785.1	Human
12LU	2007	Luebo	Democratic Republic of Congo	5°21'00.0"S	21°25'00.0"E	KC242786.1	Human
13LU	2007	Luebo	Democratic Republic of Congo	5°21'00.0"S	21°25'00.0"E	KC242787.1	Human
14LU	2007	Luebo	Democratic Republic of Congo	5°21'00.0"S	21°25'00.0"E	KC242788.1	Human
15LU	2007	Luebo	Democratic Republic of Congo	5°21'00.0"S	21°25'00.0"E	KC242789.1	Human
16LU	2007	Luebo	Democratic Republic of Congo	5°21'00.0"S	21°25'00.0"E	KC242790.1	Human
7LU	2008	Luebo	Democratic Republic of Congo	5°21'00.0"S	21°25'00.0"E	HQ613402.1	Human
5GU	2014	Gueckedou	Guinea	8°33'60.0"N	10°07'59.9"W	KJ660347.1	Human
28GU	2014	Gueckedou	Guinea	8°34'00.0"N	10°07'60.0"W	KJ660348.1	Human
29KS	2014	Kissidougou	Guinea	9°11'00.1"N	10°05'60.0"W	KJ660346.1	Human
30KA	2014	Kailahun	Sierra Leone	8°16'38.0"N	10°34'26.0"W	KM233118.1	Human
41KA	2014	Kailahun	Sierra Leone	8°16'38.0"N	10°34'26.0"W	KM233107.1	Human
52KA	2014	Kailahun	Sierra Leone	8°16'38.0"N	10°34'26.0"W	KM233096.1	Human
63KA	2014	Kailahun	Sierra Leone	8°16'38.0"N	10°34'26.0"W	KM233085.1	Human
74KA	2014	Kailahun	Sierra Leone	8°16'38.0"N	10°34'26.0"W	KM233074.1	Human
85KA	2014	Kailahun	Sierra Leone	8°16'38.0"N	10°34'26.0"W	KM233063.1	Human
96KA	2014	Kailahun	Sierra Leone	8°16'38.0"N	10°34'26.0"W	KM233052.1	Human
107KA	2014	Kailahun	Sierra Leone	8°16'38.0"N	10°34'26.0"W	KM233041.1	Human
119KA	2014	Kailahun	Sierra Leone	8°16'38.0"N	10°34'26.0"W	KM034558.1	Human
35KA	2014	Kailahun	Sierra Leone	8°16'38.0"N	10°34'26.0"W	KM233113.1	Human
45KA	2014	Kailahun	Sierra Leone	8°16'38.0"N	10°34'26.0"W	KM233103.1	Human
56KA	2014	Kailahun	Sierra Leone	8°16'38.0"N	10°34'26.0"W	KM233092.1	Human
65KA	2014	Kailahun	Sierra Leone	8°16'38.0"N	10°34'26.0"W	KM233083.1	Human
75KA	2014	Kailahun	Sierra Leone	8°16'38.0"N	10°34'26.0"W	KM233073.1	Human
86KA	2014	Kailahun	Sierra Leone	8°16'38.0"N	10°34'26.0"W	KM233062.1	Human
98KA	2014	Kailahun	Sierra Leone	8°16'38.0"N	10°34'26.0"W	KM233050.1	Human
110KA	2014	Kailahun	Sierra Leone	8°16'38.0"N	10°34'26.0"W	KM233038.1	Human
125KA	2014	Kailahun	Sierra Leone	8°16'38.0"N	10°34'26.0"W	KM034552.1	Human
128KA	2014	Kailahun	Sierra Leone	8°16'38.0"N	10°34'26.0"W	KM034549.1	Human

Supplementary Table 2. Estimates of average codon-based evolutionary diversity among sequence pairs within groups and codon-based test for diversifying selection.

Group[a]	Year	dN[b]	dS[c]	dN/dS[d]	Z-test[e]
I	1976-77	1.14 (0.66)	0 (0)	-	$p = 0.045$
II	1994-96	4.18 (1.18)	6.69 (1.65)	0.62	$p = 1$
III	2001-03	3.33 (1.08)	0.81 (0.45)	4.12	$p = 0.31$
IV	2002-05	5.48 (1.53)	3.76 (1.14)	1.46	$p = 1$
V	2007-08	0 (0)	0.44 (0.30)	0.00	$p = 1$
VI	2014	1.08 (0.48)	0.49 (0.33)	2.18	$p = 1$

a.) Grouping of sequences based on epidemic waves inferred from phylogenetic and epidemiological data. Groups correspond to clades labeled on the Bayesian maximum clade credibility phylogeny in Figure 1a.

b.) The number of nonsynonymous (dN) differences per sequence from averaging over all sequence pairs within groups and standard errors from 1000 bootstrap replicates.

c.) The number of synonymous (dS) differences per sequence from averaging over all sequence pairs within groups and standard errors from 1000 bootstrap replicates.

d.) The ratio of nonsynonymous (dN) to synonymous (dS) substitutions among codon pairs within groups.

e.) Codon-based Z-test for diversifying selection among sequence pairs within groups. The probability (p) of rejecting the null hypothesis of strict neutrality (dN = dS) in favor of the alternative hypothesis (dN > dS). Analyses were conducted using the Nei-Gojobori method with variance estimated using 1000 bootstrap replicates

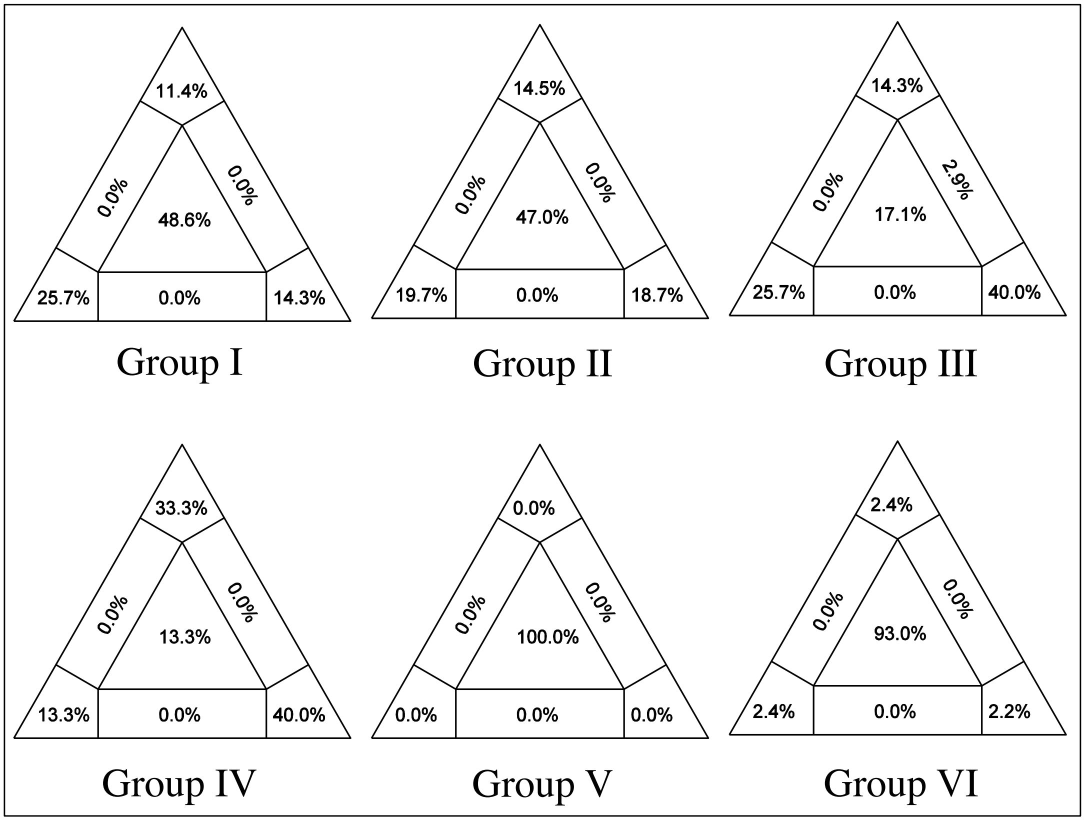

Supplementary Fig. S1. **Likelihood-mapping analysis of groups of discrete epidemics.**

The percentages of likelihoods of the three possible unrooted trees for a set of four sequences (quartets) selected randomly from the data set: dots close to the corners or the sides represent, respectively, tree-like, or network-like phylogenetic signal in the data. The central area of the likelihood map, inside the map, represents star-like signal. The percentage of dots in the central area is given at the basis of each map.

Supplementary Video 1. Discrete phylogeography of Bayesian maximum clade credibility tree and EBOV outbreak geographic locations.

Migration events of EBOV epidemic waves visualized using SPREAD v1.0.6 and mapped with ArcGIS v10.1. Migration events with Bayes Factor greater than six are displayed.
